# Paracetamol-Induced Glutathione Consumption: Is There a Link With Severe COVID-19 Illness?

**DOI:** 10.3389/fphar.2020.579944

**Published:** 2020-10-07

**Authors:** Piero Sestili, Carmela Fimognari

**Affiliations:** ^1^ Department of Biomolecular Sciences (DISB), Università degli Studi di Urbino Carlo Bo, Urbino, Italy; ^2^ Dipartimento di Scienze per la Qualità della Vita, Università degli Studi di Bologna, Rimini, Italy

**Keywords:** COVID-19, paracetamol, non-steroidal anti-inflammatory drugs, inflammation, oxidative damage, glutathione, antioxidant, risk factors

## Abstract

COVID-19 pandemic is posing an unprecedented sanitary threat: antiviral and host-directed medications to treat the disease are urgently needed. A great effort has been paid to find drugs and treatments for hospitalized, severely ill patients. However, medications used for the domiciliary management of early symptoms, notwithstanding their importance, have not been and are not presently regarded with the same attention and seriousness. In analogy with other airways viral infections, COVID-19 patients in the early phase require specific antivirals (still lacking) and non-etiotropic drugs to lower pain, fever, and control inflammation. Non-steroidal anti-inflammatory drugs (NSAIDs) and paracetamol (PAC) are widely used as non-etiotropic agents in common airways viral infections and hence are both theoretically repurposable for COVID-19. However, a warning from some research reports and National Authorities raised NSAIDs safety concerns because of the supposed induction of angiotensin-converting enzyme 2 (ACE2) levels (the receptor used by SARS-CoV2 to enter host airways cells), the increased risk of bacterial superinfections and masking of disease symptoms. As a consequence, the use of NSAIDs was, and is still, discouraged while the alternative adoption of paracetamol is still preferred. On the basis of novel data and hypothesis on the possible role of scarce glutathione (GSH) levels in the exacerbation of COVID-19 and of the GSH depleting activity of PAC, this commentary raises the question of whether PAC may be the better choice.

## Introduction

COVID-19 pandemic is posing an unprecedented sanitary threat. In the absence of specific vaccines and anti-SARS-CoV-2 drugs, medicines that may reduce the severity of the disease and limit the high number of fatalities are urgently needed. To this end, many drugs have been repurposed, including tocilizumab, sarilumab, heparin, chloroquine, ivermectin, sarilumab, chromones (Sestili and Stocchi, 2020).

Most of the effort has been so far devoted to the identification of medications capable to revert the worst and life-threatening complications of COVID-19, namely cytokine storm and hypercoagulation (Kowalewski et al., 2020; Poggiali et al., 2020). Surprisingly, however, poor attention has been paid by national and international health authorities to the development of common guidelines to treat COVID-19 in the early phases, i.e. stage 1 to stage 2A (Siddiqi and Mehra, 2020).

This crucial phase of the malady, in analogy with other viral infections and to a widely accepted pharmacological praxis, should be timely treated either with etiotropic drugs (which in COVID-19 case are still lacking) as well as with host-directed agents to manage/prevent the symptoms (Kaufmann et al., 2018). In particular, since the pivotal role of inflammation in COVID-19 life threatening complications had been identified shortly after the outbreak in Wuhan, particular attention should have been paid to identify the most active and appropriate anti-inflammatory medications, and recommend their prescription at the presentation of the early symptoms to prevent their progression (Sestili and Stocchi, 2020). Despite this simple reasoning Health Authorities rather posed a number of peremptory but questionable warnings on the early use of a wide number of anti-inflammatory drugs (Sestili and Stocchi, 2020) such as corticosteroids for their immunosuppressive activity (Veronese et al., 2020) and non‐steroidal anti‐inflammatory drugs (NSAIDs).

## NSAIDs, Paracetamol, and COVID-19

In March 2020, ibuprofen and NSAIDs were discouraged because of the alert by Micallef et al. on the supposed aggravation of COVID-19 (Micallef et al., 2020a; Micallef et al., 2020b). This warning was grounded on a wide number of studies reviewed in (Micallef et al., 2020b) including a recent report of the French Pharmacovigilance Network conducted in 2019 (Crpv De Tours, CRPV de Marseille, 2019) showing that the use of NSAIDs (even if given for short times and/or associated to antibiotics) for the treatment of fever and non-rheumatic pain (which may be indicative of an underlying infection) increases the risk of severe bacterial complications, particularly of the lungs, and on the supposed ibuprofen-induced A angiotensin-converting enzyme 2 (ACE2) overexpression. Based on these arguments, it was concluded that symptomatic treatment with NSAIDs for non-severe symptoms (fever, pain, or myalgia) is not to be recommended. Therefore, with the emergence of SARS-CoV-2 pandemic, the warning of precaution on NSAIDs became “*more topical than ever*” (Micallef et al., 2020b). Three months later a commentary by different Authors hypothesized that NSAIDs could also increase the risk of thrombosis, acute respiratory distress syndrome (ARDS), and acute renal failure in COVID-19 patients (Cumhur Cure et al., 2020).

The French Ministry of Health Olivier Veran embracing Micallef’s hypothesis, warned the public to avoid NSAIDs like ibuprofen in case of SARS-CoV-2 infection (Day, 2020); the Royal College of Obstetricians, Gynaecologists in the UK (Day, 2020) and the Italian Society of Pharmacology on April 29 (Capuano et al., 2020) agreed with this view. Prompted by these authoritative positions and in compliance to a precautionary principle, the Health Authorities of France, UK, Italy, and many other countries adopted the warning on NSAIDs recommendation. Meanwhile, the European Medicines Agency (EMA) Safety Committee decided to review all available data in the context of a safety signal procedure to verify the validity of the warning and see if any additional measure was required (Capuano et al., 2020). To date, EMA review is still ongoing.

On the other hand, the warning was immediately questioned (Moore et al., 2020; Rothuizen et al., 2020) and few months later a study on the association between routine use of NSAIDs and outcomes in hospitalized COVID-19 patients drawn completely different conclusions (Bruce et al., 2020).

Indeed, a closer view to NSAIDs pharmacodynamics might lead to opposite conclusions on their therapeutic value in SARS-CoV-2 infection. Indeed NSAIDs, by virtue of their renown anti-inflammatory and antiplatelet (especially aspirin) activities could be beneficial for both the early control of inflammation and the prevention of thromboembolism, thus theoretically limiting COVID-19 progression in a bimodal pattern. Ibuprofen, in particular, possesses Nf-kB inhibitory activity that may further help reducing excess inflammation/cytokine release in COVID-19 patients (Smart and Fawkes, 2020). Moreover, as pointed by Martins-Filho et al. (2020) in a commentary published at the beginning of June 2020 “*there is no evidence supporting the association between Ibuprofen and increased risk of severity of COVID-19*,” a position strengthened by a systematic review appeared 2 months later (Vaja et al.). Along the same line, accumulating evidence is drastically scaling back the supposed role of ACE2 upregulation by renin-angiotensin inhibitors in COVID-19 severity (Danser et al., 2020) which, according to some Authors’ view, might even be beneficial (Verdecchia et al., 2020).

Several studies are accumulating and reappraising the importance of NSAIDs in COVID-19 management (Bruce et al., 2020; Kutti Sridharan et al., 2020; Smart and Fawkes, 2020; Zolk et al., 2020); interestingly two *in silico* screenings independently identified the COX-II inhibitor celecoxib as a potential anti-SARS-CoV-2 drug, and a very recent study still under peer review reports its beneficial effects plus famotidine in hospitalized patients (Tomera et al., 2020).

Despite this accumulating evidence, paracetamol (PAC) was suggested as a safer and recommendable alternative for the early and domiciliary management of pain and fever in COVID-19 patients. Notably, PAC is a particular NSAIDs with no or negligible anti-inflammatory and antiplatelet activity (Driver et al., 2019); despite lacking these potentially valuable activities, it is the only drug that has been continuously used for the timely and domiciliary management of COVID-19 without undergoing any safety evaluation although its adverse effects could even increase depending on specific conditions, particularly those occurring in at risk COVID-19 population. Indeed, as it will be discussed below, serious concerns had been repeatedly raised on the actual PAC safety (Brune et al., 2015; Roberts et al., 2016),

## Glutathione Levels and COVID-19

An interesting breakthrough in the comprehension of COVID-19 pathogenesis may derive from three independent articles published between April and May 2020 pointing to the importance of reduced glutathione (GSH) cellular levels and integrity of the related antioxidant routes in COVID-19 pathogenesis (Aydemir and Ulusu, 2020; Polonikov, 2020; Saadat, 2020),

The first article is a commentary which proposes glucose-6 phosphate dehydrogenase (G6PD) deficiency as a factor contributing to COVID-19 morbidity and mortality (Aydemir and Ulusu, 2020). According to the Authors’ view, G6PD deficiency results in a parallel deficit in GSH levels and antioxidant activity, which in turn causes a lower capacity of the patient to overcome SARS-CoV-2 infection. The second one, is a human ecologic study proposing the hypothesis for a correlation between the glutathione S-transferase T1 (GSTT1) polymorphism and the outcome of COVID-19 (Saadat, 2020). Using univariate and multivariate analyses, the GSTT1 and GSTM1 *null* genotypes, known to be associated to an increased risk of several oxidative stress-related multifactorial diseases (Bolt and Thier, 2006), were also found more prone to COVID-19. The third article is a thoughtful viewpoint where the Author comments on the importance of antioxidant defense integrity in viral infections and proposes the hypothesis that low GSH levels may have a pathogenetic role of in COVID-19, especially in the progression toward the more aggressive presentation of the disease (Polonikov, 2020).

Over the following months further articles strengthening this view have been published: a PubMed search with the terms “COVID-19 AND glutathione” retrieved 17 records since June 1 to September 1, 2020. Among them one by De Flora et al. (2020)-who published pioneer studies on the pharmacological relevance of the GSH precursor N-acetylcysteine (NAC) in the 80s and 90s—stresses the need for thiols supplementation for both the prevention and treatment of COVID-19.

GSH, an abundant tripeptidyl molecule, contributes to the body and lung health status (Cantin and Begin, 1991) and plays pivotal roles in protecting cells against oxidative stress-induced cellular damage, in detoxifying xenobiotics and drug metabolism (Cantoni et al., 1996); decreased GSH levels are associated with the common features of aging as well as of a wide range of pathological conditions (Homma and Fujii, 2015), comorbidities, smoking habit which, intriguingly, represent the major risk factors for COVID-19.

Resistance to viral diseases positively correlates with the extent of GSH stores (Khomich et al., 2018). Higher levels of GSH have been associated with better individual’s responsiveness to viral infections (De Flora et al., 1997; Lee, 2018): in particular, GSH is known to protect host immune cells operating in oxidative stressing environments and contributes to their optimal functioning. Reactive oxygen species (ROS)-induced alterations of the immune response has been proposed as a key player in COVID-19 pathogenesis and antioxidant intervention with NAC recommended as a preventive and therapeutic strategy (De Flora et al., 2020; Schönrich et al., 2020).

Interestingly, preventive supplementation of NAC significantly reduced the incidence of clinically apparent influenza, especially in higher risk elderly population (De Flora et al., 1997). This effect may also depend on the GSH-induced inhibition of various respiratory viruses’ replication, an effect which is thought to prevent increased viral loads and the subsequent massive release of inflammatory cells into the lung. i.e. cytokine storm (Palamara et al., 1996; Nencioni et al., 2003). To this regard, GSH may also have direct anti-SARS-CoV-2 potential: indeed a computational study indicates that the binding of spike protein to ACE2 is maximal when ACE2-sulfur groups are in the form of disulfides and impaired when fully reduced to thiols: hence a prooxidant environment with low levels of GSH would favor viruses cellular entry (Hati and Bhattacharyya, 2020).

The deficiency of GSH in the alveolar fluid in ARDS patients was found to correlate with the increased ROS-mediated lung cell injury and inflammation (Pacht et al., 1991; Soltan-Sharifi et al., 2007): Soltan-Sharifi also reported that supplemental NAC resulted in the prevention of this aggravating condition (Soltan-Sharifi et al., 2007). Incidentally, both ARDS and cytokine storm characterize the last COVID-19 stages (Siddiqi and Mehra, 2020).

GSH levels positively correlate with those of active vitamin D (Jain et al., 2014), whose deficiency has been shown to play a detrimental role in COVID-19 (Grant et al., 2020; Jain and Parsanathan, 2020; Meltzer et al., 2020).

GSH deficiency results in the activation of von Willebrand Factor (Ibrahim et al., 2004) and in the accumulation of ROS, which affect clotting and platelet activation, impair endothelial function, and predispose to the risk of thrombotic events (Violi et al., 2017): notably, hypercoagulation is a prominent life-threatening complication in COVID-19 patients (Giardini et al., 2020).

Notably, low GSH plasma levels have been identified along with three other clinical indicators (age, CD3 ratio, and total protein) as a predictor of the severe/critical symptoms of COVID-19 infected patients (Sun et al., 2020).

**Figure 1:**
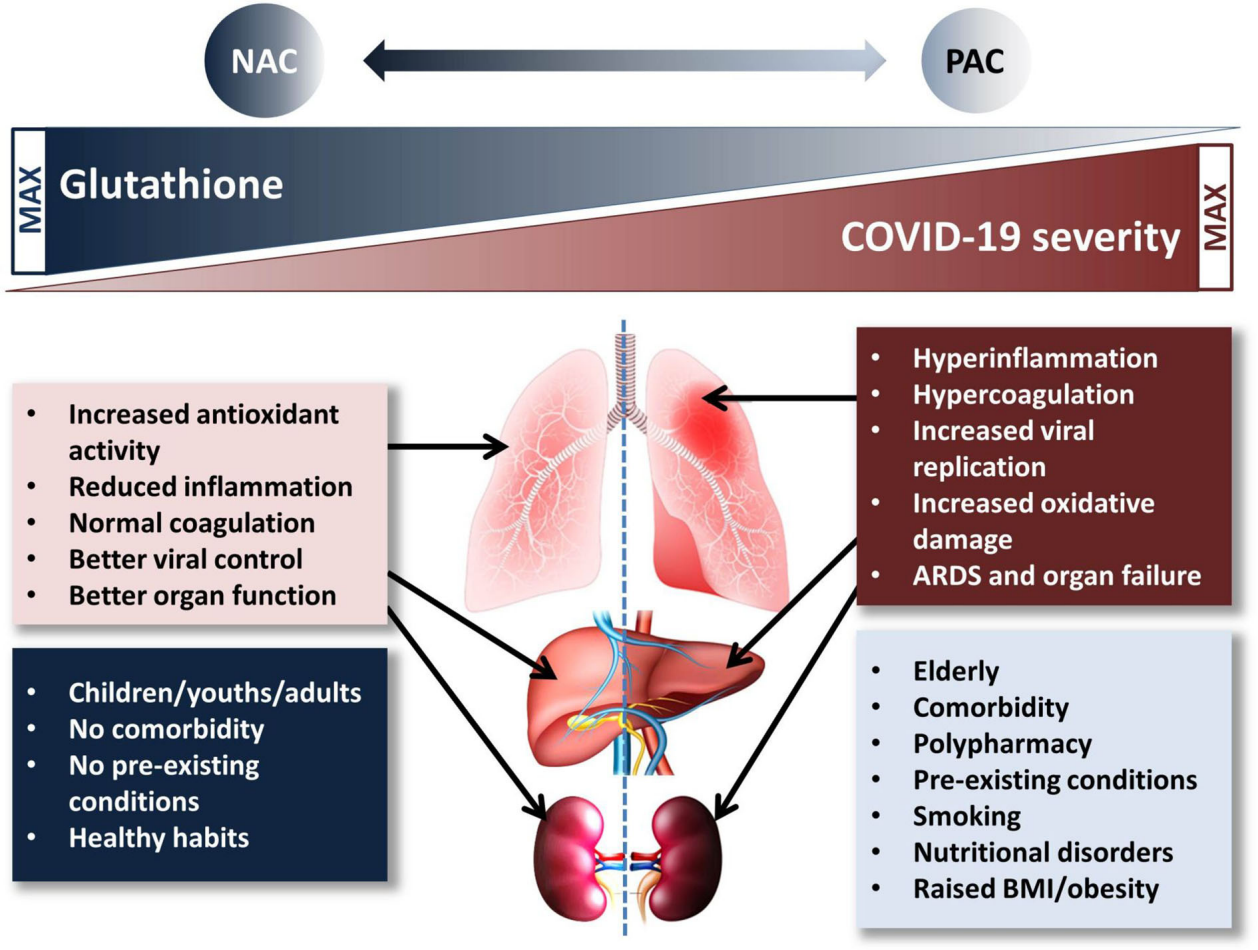
Proposed mechanism of the glutathione-paracetamol-COVID-19 interactions. Low risk population groups (blue box) have normal/high GSH levels which contribute to the beneficial effects in COVID-19 (pink box). On the contrary, high risk population groups (pale blue box) are characterized by low GSH levels which cannot help modulating the deleterious events causing (brick-red box) severe COVID-19. N-acetylcysteine supplementation increases, while paracetamol may reduce GSH availability and negatively impact on lung, liver, and kidney function, especially in *per se* low-GSH subjects. ARDS, acute respiratory distress syndrome; BMI, body mass index; NAC, N-acetylcysteine; PAC, paracetamol.

All the above evidences seem to have a clinical relevance: Polonikov, studying four moderate-severe COVID-19 cases, found that while the three patients with normal/high plasma levels of GSH recovered rapidly, the one with low GSH levels, high plasma ROS and ROS/GSH ratio experienced the most severe illness and, at the date of publication, was still sick (Polonikov, 2020). In another case report, two COVID-19 pneumonia patients were successfully treated with high doses of supplemental intravenous glutathione and oral NAC (Horowitz et al., 2020); finally, Ibrahim et al. reported the case of a group of nine severely ill patients successfully treated with NAC (Ibrahim et al., 2020). Notably, these three independent reports—although referring to only fifteen cases—reciprocally strengthen each other in highlighting the relevance of poor GSH levels in COVID-19 clinical progression as well as the importance of maintaining/repleting GSH pools as a countermeasure against SARS-CoV2 virulence. To this end, it is important noting that the major risk factors for severe COVID-19 illness are aging, comorbidities, smoking habit, all characterized by intrinsically low antioxidant capacity and high ROS/GSH ratios (Polonikov, 2020) (see **Figure 1**). Hence, according to precautionary principle any condition potentially leading to further depletion of GSH stores should be carefully avoided.

## Paracetamol for the Early Management of COVID-19: A Critical Viewpoint

As discussed above, a precautionary principle regarding the risk of bacterial superinfections and of ACE2 overexpression was the main reason for the decision to discourage the use of NSAIDs (Capuano et al., 2020; FitzGerald, 2020; Micallef et al., 2020a; Micallef et al., 2020b; Soeiro et al., 2020) in favor of PAC, reputed as a safer drug.

However, contrary to this opinion, we believe that in the specific case of COVID-19 it is of paramount importance taking due account of the fact that PAC and its metabolites decrease GSH levels, also when given at relatively low doses in healthy volunteers (Burgunder et al., 1989; Nuttall et al., 2003; Pujos-Guillot et al., 2012; Stahl et al., 2015).

Although the drop in hepatic or renal GSH is the most toxicologically relevant interaction (see also below), plasma GSH, free cysteine (Burgunder et al., 1989), and antioxidant capacity (Nuttall et al., 2003) were significantly reduced after a single 2 g PAC administration or 14 days of therapeutic doses of PAC in human volunteers, respectively; 3 g PAC for 14 days in older people led to a significant reduction of sulfur amino acids (Pujos-Guillot et al., 2012). It is of worth that PAC plasma levels can even increase above the expected concentrations exacerbating thiol consumption under conditions of gut dysbiosis (Mukhtar et al., 2019), another common status in COVID-19 at risk population (Aktas and Aslim, 2020). Furthermore, clinically attainable concentrations of PAC have been shown to decrease *in vitro* intracellular GSH in human pulmonary macrophages, type II pneumocytes, and lymphocytes (Estévez et al., 1994; Dimova et al., 2005). Notably, the depletion of GSH in airway mucosa is considered as the most biologically plausible mechanism of the established epidemiologic association between PAC use and asthma prevalence/severity in children and adults (Shaheen et al., 2000; McBride, 2011), implicitly suggesting that GSH depletion may take place also in other clinical settings.

Oxidized PAC-quinone imine metabolites have also been shown to form GSH-conjugates which inhibit glutathione reductase (GR): the decreased activity of GR hampers the detoxification and antioxidant capacity of the GSH-GSSG cycle, further aggravating the pro-oxidative status in the cell (Nýdlová et al., 2014).

From a different toxicological perspective, a study by Klopčič et al. indicates that PAC, in the absence of adequate, physiological levels of GSH, may give rise to genotoxic quinone imine metabolites (Klopčič et al., 2015). As a consequence, although clinical application of PAC may be considered as safe, in the case of severely depleted GSH levels PAC should be administered with caution, especially in subjects with severe GSH depletion who, again, are those at higher risk of developing severe COVID-19 disease.

The production of quinone imine metabolite is the primary responsible for PAC liver and kidney toxicity. Ninety-seven percent of drug-induced acute liver failure have been ascribed to PAC; liver enzyme alterations are very frequent in PAC treated patients, even at routine dosage; the maintenance of patients’ liver and kidney function is obviously important for the body’s ability to react to infections, including COVID-19. Importantly, recent reports showed that about 2–11% of patients with COVID-19 had underlying chronic liver disease (Jothimani et al., 2020) and that abnormal liver function is rather common in the course of the malady. These effects, deriving either by the direct action of the virus or by the use of drugs (i.e. lopinavir and ritonavir), showed an association with the progression of liver damage in severe cases (Ali, 2020). As to kidneys, their importance in COVID-19 is twofold since their function is not only important for patient’s recovery, but they are also a target organ of SARS-CoV-2 (Farouk et al., 2020). Hence any condition potentially impairing liver and kidney status—including extensive PAC use—should be carefully avoided, especially in aged and comorbid population where these organs are often impaired due to pre-existing conditions.

The above and other reports led Roberts et al. to the following conclusions with regard to PAC actual safety: “we believe the true risk of paracetamol prescription to be higher than that currently perceived in the clinical community. Given its high usage and availability as an over-the-counter analgesic, a systematic review of paracetamol’s efficacy and tolerability in individual conditions is warranted” (Roberts et al., 2016).

On the whole, although there is no direct evidence in COVID-19 patients, PAC is likely to promote GSH depletion, especially in those population groups at higher risk (Mast et al., 2018).

The following considerations further strengthen the criticism toward the use of PAC as a safer alternative to NSAID:

1. PAC has been preferred to NSAIDs and steroids for the symptomatic and domiciliary management of the early stages of COVID-19, a choice grounded of the precautionary principle (but still theoretical in this specific case) principle “*primum non nocere*” (Micallef et al., 2020a).
2. Counterintuitively, however, the same precautionary principle has not been applied to PAC itself, and the risks of developing severe COVID-19 associated to the reduction of GSH might be far higher than the benefits derived from discouraging the use of NSAIDs or steroids.
3. In addition, PAC has the capacity to reduce fever and pain as well as NSAIDs (Messika et al., 2014), and may equally mask the symptoms delaying the objective grading of the disease, but it lacks the NSAIDs anti-inflammatory and antiplatelet activities that might be fundamental in containing COVID-19 exacerbation (Giardini et al., 2020). Although merely anecdotal, there is wide and transnational evidence of patients left at home with mild symptoms for more than a week receiving only PAC until their worsening conditions required hospitalization and, not rarely, admission to intensive care units.
4. The routinary use of PAC in at risk categories, along with their intrinsically frail conditions, may have further worsen the scarcity of GSH, especially in western countries where PAC consumption is particularly high. Such a situation may have rendered this group of population even more susceptible to SARS-CoV2 at the time of its spreading. To this end a merely speculative but intriguing hypothesis is that PAC adoption might have contributed to the high virulence of COVID-19 observed in many EU countries and USA. Notably, in most countries PAC is freely sold as an OTC drug, raising the risk of unintentional abuse and increased adverse effects (Sansgiry et al., 2017).
5. No answer can be given to the above open questions because PAC efficacy/adverse effects, unlike most of the drugs repositioned for COVID-19 therapy, have not yet been evaluated in controlled clinical trials or analyzed through retrospective analyses. These trials, as well as studies aimed at determining the levels of GSH in the plasma of PAC-treated *vs* -untreated COVID-19 patients should be encouraged.

## Conclusive Remarks

The preferential use of PAC in COVID-19 as a safer alternative to NSAIDs should be carefully reconsidered and NSAIDs use eventually reappraised. Finally, countries experiencing a new rise of SARS-CoV-2 positive cases such as the four major EU nations and UK, should promote the development of more rational treatment guidelines for COVID-19, taking due account of the above facts and considerations to avoid that the same mistake—if concerns on PAC are ascertained—may be repeated in the next few months.

## Data Availability Statement

Publicly available datasets were analyzed in this study. This data can be found here: https://pubmed.ncbi.nlm.nih.gov/.

## Author Contributions

PS: conceptualization and writing. CF: bibliographic search, revision of the text. All authors contributed to the article and approved the submitted version.

## Funding

Institutional and unconditional University funding.

## Conflict of Interest

The authors declare that the research was conducted in the absence of any commercial or financial relationships that could be construed as a potential conflict of interest.
